# Clinical implications of granulomatous inflammation detected by endobronchial ultrasound transbronchial needle aspiration in patients with suspected cancer recurrence in the mediastinum

**DOI:** 10.1186/1749-8090-3-8

**Published:** 2008-02-25

**Authors:** Marcus P Kennedy, Carlos A Jimenez, Ashwini D Mhatre, Rodolfo C Morice, Georgie A Eapen

**Affiliations:** 1 Department of Pulmonary Medicine, University of Texas MD Anderson Cancer Center, Houston, Texas, USA

## Abstract

**Background:**

Granulomatous inflammation has been previously reported in association with cancer. Endobronchial ultrasound guided transbronchial needle aspiration (EBUS-TBNA) is a new minimally invasive test for investigating mediastinal lymphadenopathy. The identification of granulomatous inflammation by EBUS-TBNA and the clinical implications of such detection in a series of patients with previously treated cancer and new mediastinal lymphadenopathy has not previously been performed.

**Methods:**

All 153 consecutive patients undergoing EBUS-TBNA in an academic cancer institution for suspected cancer in the mediastinum (mediastinal lymphadenopathy by CT imaging) were reviewed. Patients with non-caseating granuloma identified by EBUS-TBNA were included.

**Results:**

EBUS-TBNA identified non-caseating granuloma in 17/153 (11%) patients. A subset of 8/153 (5.2%) had sarcoid like lymphadenopathy mimicking cancer recurrence (5/5 PET positive). Another 8/153 (5.2%) patients with new mediastinal lymphadenopathy and no prior history of cancer had a clinical syndrome consistent with sarcoidosis. One other patient with a history of breast cancer was diagnosed with non-tuberculous mycobacteria infection. No patient required mediastinoscopy and there were no complications.

**Conclusion:**

In an academic cancer institute, at least 5% of patients undergoing EBUS-TBNA have sarcoid-like lymphadenopathy mimicking cancer recurrence. Further studies to define the precise etiology, natural history and prognosis of this phenomenon are warranted.

## Background

In patients with a history of cancer, the onset of mediastinal adenopathy often heralds a recurrence of their malignancy. However, not all mediastinal adenopathy is due to cancer recurrence and lymph node sampling is warranted. Endobronchial ultrasound transbronchial needle aspiration (EBUS-TBNA) allows real time assessment and biopsy of mediastinal lymph nodes and therefore often obviates the need for mediastinoscopy [1,2]. The utility of EBUS-TBNA in the mediastinal nodal staging of lung cancer has been defined [3]. More recently, the ability of EBUS-TBNA to identify granuloma in the work-up of patients with mediastinal adenopathy secondary to suspected sarcoidosis has been reported [4]. Since the beginning of the last century, "local sarcoid reactions" and "sarcoid like lymphadenopathy" with pulmonary and mediastinal involvement have been described in patients with cancer [5-7]. In this report, we describe a group of patients in whom EBUS-TBNA detected granulomatous inflammation in suspected cancer recurrence and examine the clinical implications of such a finding.

## Methods

### Patients

We reviewed all EBUS-TBNA performed at our institution from August 2005 to September 2006 for undiagnosed mediastinal adenopathy. Patients referred for mediastinal staging were specifically excluded from further analysis. All other patients in whom EBUS-TBNA identified non-caseating granulomatous inflammation were analyzed. The diagnosis of sarcoidosis or sarcoid like lymphadenopathy was made if clinico-radiological findings were supported by histopathologic findings from EBUS-TBNA along with appropriate exclusion of other granulomatous diseases (a composite of clinical history, follow-up and laboratory results including tissue staining for fungi and acid fast bacilli (AFB), fungal and mycobacterial cultures and serum fungal antibody titers).

### Procedure

All of the EBUS -TBNA were performed by interventional pulmonology attendings (n = 3) with or without supervised fellows. Under general anesthesia with ventilation via a laryngeal mask airway, conventional flexible bronchoscopy (model BF-T160 bronchoscope, Olympus, Japan) was first performed to examine the tracheobronchial tree. Thereafter, EBUS-TBNA using a linear array ultrasonic bronchscope (Olympus XBF-UC 160F) with dedicated 22-gauge needle (NA-202C Olympus ltd.) was performed with ultrasonic examination of mediastinal and hilar lymph nodes in a systematic fashion and subsequent transbronchial needle aspiration [8]. All lymph nodes greater than 5 mm in short axis diameter were sampled. A minimum of two separate passes of the 22-gauge needle into each node was performed. The aspirated material was smeared onto glass slides and separately labeled. Smears were air dried as well as fixed in Carnoy's solution (6 parts EtOH (absolute or 95%) 3 parts chloroform 1 part glacial acetic acid). Additional material was aspirated into RPMI (Sigma-Aldrich, St. Louis, MO) and analyzed using cytospin or cellblocks. Air dried smears were stained using Diff-Quik stain (American Scientific Products; McGaw Park, IL) and fixed specimens were stained with Papanicolaou staining and examined by an on-site cytologist to ensure adequate cellular material in the specimen. If adequate tissue was not identified by on site cytology after five passes, no additional passes were carried out at that station. Histologic core samples were not obtained in any of the patients. Our institutional review board approved this analysis.

## Results

**Figure 1:**
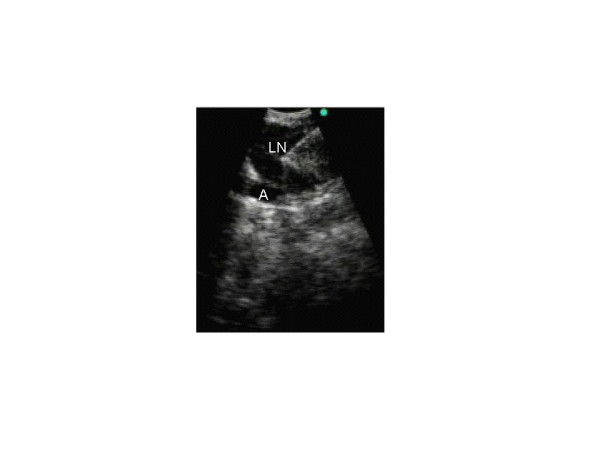
Endobronchial ultrasound image depicting transbronchial needle aspiration of a left hilar lymph node 11L (LN) overlying left interlobar artery (A) in a patient with history of breast cancer and new hilar adenopathy.

All 153 consecutive patients referred for EBUS-TBNA for mediastinal lymphadenopathy identified by CT imaging were included. No cancer history was identified in 38 (24.8%). We identified 17 patients (11%) in whom EBUS-TBNA identified non-caseating granuloma without any evidence of malignancy (Figure 1). Cytological analysis of EBUS-TBNA samples did not identify granulomatous inflammation and cancer in the same node or different nodal stations during the same procedure in any of the patients.

### Granulomatous inflammation by EBUS-TBNA in patients with a prior history of cancer (n = 9)

A subset of nine patients (6%) had a prior history of cancer and is highlighted in Table 1. All had new mediastinal lymphadenopathy suspicious for cancer recurrence and EBUS-TBNA identified granulomatous inflammation without any evidence of cancer. No patient had a prior history of granulomatous disease or infection. A PET scan had been performed in 5 patients prior to EBUS-TBNA and the enlarged mediastinal lymph nodes were positive in all. Upon review of prior cancer treatment, five of the eight patients received chemotherapy, including imatinib, and docetaxel in two patients each.

**Table 1:** Clinical, radiographic and demographic features of nine patients with suspected cancer recurrence in whom endobronchial ultrasound transbronchial needle aspiration detected granulomatous inflammation.

**Age Sex Race**	**Oncological History Remission (yrs since initial diagnosis)**	**Chemotherapy**	**Lymph node Characteristics**			**Granuloma at other sites**	Microbiologic Studies*			**Treatment**	**Follow-up (months)**
							
			**Station [8]**	**Size mm**	**PET +ve**		**Culture (n)**	**Staining**	**Serology**		
1 56F C	Breast Cancer Neadjuvant Chemotherapy	Paclitaxel Lapatinib	7 11R	14 7	-	No	P	N	N	MAC Infection Rifabutin Ethambutol Clarithromycin	Radiographical and clinical improvement on antimycobacterial therapy
2 28F C	Melanoma (2)	No	4R 7	7 13	-	No	N (1)	N	-	None	No symptoms (11)
3 49M H	Hodgkin's Lymphoma Stage 1a (8)	No	11R	14	Yes	No	N (2)	N	-	None	No symptoms Node PET uptake and CT size reduced (6)
4 56F AA	GI Stromal Tumor (1)	Imatimib	4R	17	-	TBBX**	N (1)	N	-	Inhaled Fluticasone	Productive Cough Progressive Pulmonary Infiltrates (12)
5 61F C	NSCLC T2N2M0 (1)	Carboplatin Docetaxel	11R	9	Yes	No	N (1)	N	-	Inhaled Fluticasone	Chronic cough Stable CT-PET (6)
6 63M C	Melanoma (4)	No	2R 7	10 11	Yes Yes	Lymphocytic Meningitis**	N (3)	N	N	Prednisone For neurosarcoidosis	Neurological Symptoms No pulmonary symptoms Node PET uptake and CT size reduced (15)
7 66M C	Multiple Myeloma (3)	Melphalan Thalidomide Lenalidomide	11R	11	Yes	EBBX** TBBX	N (2)	N	N	None	No symptoms Stable CT-PET (8)
8 68F AA	Endometrial Cancer (2)	Docetaxel Carboplatin Imatimib	7 11R	13 10	Yes Yes	No**	N (1)	N	-	None	No symptoms Stable CT-PET Intraabdominal cancer Recurrence (9)
9 77M C	Colorectal Cancer (3)	Cisplatin Capecitabine	7	10	-	No**	N (2)	N	-	None	No symptoms Stable CT (10)

* Fungal and mycobacterial cultures (bronchoalveolar lavage or bronchial wash or sputum), tissue staining for fungi and acid fast bacilli (AFB), and serum fungal antibody titers.

** Pulmonary parenchymal involvement on high resolution CT.

AA = African American, AFB = acid fast bacilli, BAL = bronchoalveolar lavage, C = Caucasian, CT = computed tomography, EBBX = endobronchial biopsy, EBUS = endobronchial ultrasound, F = female, GI = gastrointestinal, H = Hispanic, M = male, MAC = *Mycobacterium avium intracellulare *infection, N = negative, P = positive, PET = positron emission tomography, TBBX = transbronchial biopsy, Yrs = years

Non-tuberculous mycobacteria infection (*Mycobacterium avium intracellulare*) was identified in one of the nine patients with symptoms responding to antimycobacterial treatment. Tissue staining for AFB organisms and fungi and respiratory cultures (average 1.6 per patient) were negative in all of the other eight patients. In all eight patients, pathological review of all the tissue available at the original time of cancer diagnosis did not reveal any evidence of granulomatous inflammation. One patient (patient 5) had evidence of mediastinal lympadenopathy at the time of original cancer diagnosis, and a mediastinoscopy at the time was positive for cancer, but negative for granuloma. However, the mediastinal adenopathy regressed with therapy and restaging scans subsequently revealed recurrent mediastinal lymphadenopathy requiring repeat sampling (EBUS-TBNA) that revealed granulomatous inflammation.

Evidence for granulomatous inflammation at other sites was also evaluated. Five patients had pulmonary parenchymal abnormalities consistent with sarcoid parenchymal disease on high resolution CT scan. Two of these patients had concomitant transbronchial lung biopsies positive for granuloma.

Confirmatory medistinoscopy was not deemed necessary for any of the patients and close radiographic follow up was instituted. Upon follow-up for an average of 10 months (range 6–15 month), two patients developed progressive cough that responded to treatment with inhaled fluticasone, while one other patient developed symptomatic lymphocytic meningitis consistent with neurosarcoidosis and was treated with systemic steroids. All other patients remained clinically and radiographically stable except for one patient who developed an intraabdominal recurrence of endometrial cancer. Interestingly however, her mediastinal adenopathy remained unchanged.

### Granulomatous inflammation by EBUS-TBNA in patients without a prior history of cancer (n = 8)

The other 8/17 patients had no prior cancer history, and underwent EBUS-TBNA to rule out a malignant cause of mediastinal lymphadenopathy. Granulomatous inflammation was found in all by EBUS TBNA and all 8 were ultimately diagnosed with systemic sarcoidosis on the basis of a compatible clinical history, granulomatous inflammation (by EBUS-TBNA and transbronchial lung biopsy in 5 patients) and adequate exclusion of other granulomatous diseases. There were no complications related to the EBUS-TBNA procedure in any of the 17 patients.

## Discussion

Consistent with the recently published study of the utility of EBUS-TBNA in the diagnosis of sarcoidosis, we have confirmed that EBUS-TBNA can identify granulomatous disease in the mediastinum [4]. However, in an academic cancer center, we identified a sub-group of patients with a prior history of cancer with new mediastinal lymphadenopathy in whom EBUS-TBNA detected granulomatous inflammation. These patients account for at least 5% of all EBUS-TBNA performed at our institution. This may be an underestimation as we only perform cytological examination of aspirated material routinely, without histologic cores that are difficult to obtain with the currently available 22 gauge needles. Furthermore, the false negative rate for granulomatous inflammation was not investigated with mediastinoscopy in our series.

Despite the fact that it has been recognized for over a century that an association between granulomatous inflammation and cancer exists, the cause of this relationship is unknown [5-7]. Many hypotheses exist such as immunological dysfunction related to cancer and sarcoidosis, a side effect of cancer therapy (although the phenomenon has been described in cancer patients treated with surgery alone [9]), and "antigenic shedding" from the tumor leading to granuloma formation [6,7]. In fact, there is no agreement on what to name the phenomenon of mediastinal and hilar adenopathy secondary to granulomatous inflammation in treated cancer patients (sarcoid like reaction, sarcoid like lymphadenopathy, pulmonary and mediastinal "sarcoidosis" or simply sarcoidosis). A detailed analysis of these hypotheses and arguments is beyond the limits of this report.

Prior reports have highlighted this phenomenon in many different cancer types, although there seems to be an over representation of patients with testicular germ cell tumors [6,7,9-12]. Characteristics of the patients reported in this manuscript such as a lack of prior history of granulomatous disease or evidence of mediastinal adenopathy at the time of cancer diagnosis, no ethnic association, varying time between cancer and sarcoid diagnosis, adenopathy distant to the primary site and varying course and response to treatment are in keeping with prior reports and differentiate this phenomenon from local sarcoid reactions in draining lymph nodes which occur at the time of primary cancer diagnosis [6,7,9-12]. Of note, two of the patients we report were treated with imatinib and two with docetaxel which both have been associated with interstitial lung disease, although neither with granulomatous inflammation [13,14].

The clinical relevance of this phenomenon, however, is clear. Attributing radiographic findings such as mediastinal lymphadenopathy without tissue confirmation as cancer recurrence can lead to unnecessary and toxic therapy [11]. In all the patients reported in this manuscript, mediastinal lymphadenopathy met criteria for cancer recurrence by CT measurement and mediastinal lymphadenopathy was considered FDG-avid in all five patients who underwent PET imaging. In practice, new onset hilar or mediastinal adenopathy is often attributed to cancer recurrence without definitive tissue confirmation. This is a situation fraught with peril as mediastinal and hilar adenopathy may be secondary to other processes such as granulomatous inflammation and not cancer recurrence. However, in patients with a prior history of cancer, mediastinoscopy, the 'gold standard' for mediastinal lymph node sampling, may be difficult to perform particularly with prior instrumentation or thoracic radiation therapy. These patients are often frail with multiple comorbidities that may preclude surgical options. With the advent of EBUS-TBNA, a minimally invasive tool is now available that allows for safe, accurate and repeated hilar and mediastinal lymph node sampling. Despite the small population studied, the fact that all patients diagnosed with granulomatous inflammation by EBUS -TBNA remained clinically stable would seem to indicate that EBUS-TBNA is able to reliably differentiate this sarcoid like mediastinal granulomatous inflammation from cancer recurrence. Furthermore, only one patient had culture evidence of a granulomatous infection, and all other patients remained clinically stable without any antibiotic therapy, again supporting a non-infectious etiology for the adenopathy. Accordingly, if granulomatous inflammation is identified by EBUS-TBNA in a patient with suspected cancer recurrence, a reasonable clinical approach would be to follow the patient radiographically without additional invasive testing, unless radiographic progression was subsequently noted.

## Conclusion

This study highlights the ability of EBUS-TBNA to detect granulomatous inflammation in patients with suspected cancer recurrence in mediastinal and hilar lymph nodes. In this era of increasing cancer survivorship, and with the improvements in sampling technology, this phenomenon of granulomatous inflammation following cancer and cancer therapy is likely to become a more commonly recognized entity and further study to define the precise etiology, natural history and prognosis is warranted.

## Competing interests

The author(s) declare that they have no competing interests.
